# IgG4-related retroperitoneal fibrosis presenting as a peripancreatic mass: a case series

**DOI:** 10.1097/MS9.0000000000002749

**Published:** 2025-01-09

**Authors:** Maaz Bin Badshah, Qaisar Ali Khan, Naima Kazi, Rabia Aslam Ansari, Ravina Verma

**Affiliations:** a Shifa International Hospital, Islamabad, Pakistan; b Khyber Teaching Hospital, MTI KTH, Peshawar, Pakistan; c Khyber Girls Medical College, Peshawar, Pakistan; d Allama Iqbal Medical College, Lahore, Pakistan; e St. George’s University School of Medicine, True Blue, Grenada

**Keywords:** case report, histopathology, IgG4-related diseases, retroperitoneal fibrosis, retroperitoneal mass

## Abstract

**Introduction:**

IgG4-related disease (IgG4-RD) is a chronic, immune-mediated disorder characterized by widespread inflammation and fibrosis, leading to potential organ dysfunction if untreated. Often underdiagnosed due to subtle and varied symptoms, the disease can affect multiple organ systems. This case series highlights two patients who were diagnosed as cases of IgG4-related retroperitoneal fibrosis.

**Methods:**

A total of two patients were included in this prospective case series who presented to the gastroenterology department of a tertiary care hospital with the same signs and symptoms and were diagnosed with IgG4-related retroperitoneal fibrosis (IgG4-RPF).

**Case summary:**

Two patients were included in our case series, aged 25 and 26 years. The chief complaints included dull, radiating epigastric pain, other symptoms include diffuse abdominal pain, intensified, accompanied by nausea, vomiting, and episodic diarrhea, and a history of B-cell lymphoproliferative disorder. Endoscopic ultrasound (EUS)-)-guided biopsies were performed, showing findings consistent with (IgG4-RPF). Both patients were started on a regimen of prednisolone, pantoprazole, and vitamin D, which they tolerated well without adverse effects. They were advised to follow up with a CT scan after one month

**Conclusion:**

IgG4-related disease (IgG4-RD) is a rare, chronic condition often presenting as retroperitoneal fibrosis (RPF) and affecting multiple organs. Serum IgG4 levels can be normal in certain cases, histopathological and radiological investigations are necessary for the correct diagnosis. Early initiation of immunosuppressive drugs are necessary for the disease control.

## Introduction

IgG4-related disease (IgG4-RD) is a chronic, immune-mediated disorder characterized by extensive inflammation and fibrosis, potentially resulting in significant organ dysfunction if not properly managed.^[1]^ First recognized as a distinct condition in the early 2000s, IgG4-RD is notable for its capacity to affect almost any organ system. Despite its broad impact, the disease is often underdiagnosed due to its subtle and varied clinical presentations. According to estimates from Japan’s Ministry of Health, Labour, and Welfare, the incidence of IgG4-RD is relatively low, ranging from 0.28 to 1.08 per 100,000 individuals, with 336 to 1,300 new cases identified annually. IgG4-RD accounts for approximately 30% to 60% of all RPF cases. Retroperitoneal fibrosis is one of the most commonly encountered subsets of IgG4-RD, affecting 3% to 19% of these patients^[2,3]^

The pathophysiology of IgG4-related disease (IgG4-RD) is characterized by the accumulation of IgG4-expressing plasma cells within affected tissues, indicating chronic activation of the adaptive immune system.^[4]^ Although the humoral immune response, evidenced by hypergammaglobulinemia and pronounced IgG4 class-switching, has been extensively studied, the precise role of IgG4 in the disease mechanism remains unclear. Unlike other fibrotic disorders such as systemic sclerosis or idiopathic pulmonary fibrosis, tissue fibrosis in IgG4-related disease (IgG4-RD) may be reversible. B cell depletion therapy has shown remarkable efficacy, highlighting a key role for B cell/T cell interactions in disease pathogenesis. Abnormal Th2/regulatory T cells, sustained by autoreactive B cells, are suspected to promote fibrosis through profibrotic cytokines, though this remains unproven. Key questions about IgG4-RD pathogenesis, including antigenic triggers and the role of IgG4 antibodies, remain unresolved, and recent studies suggest innate immunity may precede adaptive responses.^[5]^ Diagnosis of IgG4-RD requires both histopathological confirmation and clinicopathological confirmation. Serological and Radiological tests have decreased sensitivity and specificity for the diagnosis of IgG4-RD. Clinically, elevated IgG4 levels at the time of diagnosis are linked to more widespread organ involvement and an increased risk of relapse. Histopathological findings include dense lymphoplasmacytic infiltration, storiform fibrosis, and obliterative phlebitis, with additional consideration given to tissue IgG4 counts and IgG4/IgG ratios.^[5,6]^

IgG4-RD can affect a wide range of organ systems, including the meninges, orbits, lacrimal glands, lungs, pancreas, kidneys, biliary system, and retroperitoneum. Patients may exhibit nonspecific symptoms that can persist for months or even years before a correct diagnosis is made, and these symptoms are often misinterpreted by healthcare providers. The disease is frequently discovered incidentally through imaging or during a pathological examination following surgery. Because of its often prolonged subclinical phase, nearly 60% of patients with IgG4-RD suffer some degree of irreversible organ damage by the time they are diagnosed.^[7]^ The natural history and prognosis have not been defined in sufficient detail. Spontaneous improvement can be seen, but the disease often recurs without treatment. Most patients respond initially to therapy with glucocorticoids, but relapses are common following discontinuation of therapy. Significant organ dysfunction may arise from uncontrolled and progressive inflammatory and fibrotic changes in affected tissues. The possibility of increased risk of malignancy in patients with IgG4-RD requires further study^[8]^

This case series aims to highlight the diagnostic challenges and clinical presentation of IgG4-related retroperitoneal fibrosis by detailing the cases of two patients who presented with similar symptoms of abdominal pain. By examining these cases, we aim to contribute to the growing body of literature on IgG4-RD, with the hope of improving recognition, diagnosis and management of this complex and often underdiagnosed condition’. This case series has been reported per the Preferred Reporting Of Case Series in Surgery (PROCESS) guideline with careful attention to transparent and comprehensive reporting.^[9]^

## Methods

Study design and setting: The study is a single-center prospective case series conducted in the gastroenterology department of a tertiary care hospital.

Participants: A total of two patients were enrolled in this study who presented from the same geographical area of Pakistan with the complaint of chronic abdominal pain. The clinical presentation, demographic data, medical history, and relevant past interventions were documented through electronic medical records (EMR) and patient interviews. Both patients were diagnosed with IgG4-related retroperitoneal fibrosis and were managed at our tertiary care facility.

### Clinical findings and timeline

The clinical evaluation was based on the patient’s presenting complaints, physical examinations, and laboratory findings. Investigations included routine blood tests, serum IgG4 levels, contrast-enhanced computed tomography (CT), and tissue biopsy. A chronological timeline was created, documenting the onset of symptoms, diagnostic procedures, and treatment interventions.

### Diagnostic approach

The diagnosis was made based on clinical and histopathological findings. A tissue biopsy confirmed the diagnosis. Radiological investigations supported the diagnosis by showing the characteristic features of retroperitoneal fibrosis.

## Results

### Case 1 presentation

A 25-year-old male with a history of B-cell lymphoproliferative disorder presented to the gastroenterology outpatient clinic with chronic abdominal pain persisting for six months. The pain was initially mild, gradually increased in intensity, and radiated to the back. Described as dull and localized to the epigastrium, the pain recently intensified, reaching 8 out of 10 on the pain scale. The patient denied any associated symptoms such as weight loss, nausea, vomiting, melena, or hematemesis.

Previously, in 2023, the patient was treated for B-cell lymphoproliferative disorder involving the peripancreatic region. The diagnosis was confirmed via a positron emission tomography (PET) scan, which showed a lobulated, fluorodeoxyglucose-avid mass just below the origin of the inferior mesenteric vessel, extending into the mesenteric region. An endoscopic ultrasound (EUS)-guided biopsy performed at that time revealed CD20-positive cells, confirming the diagnosis of B-cell lymphoproliferative disorder.

**Figure 1:**
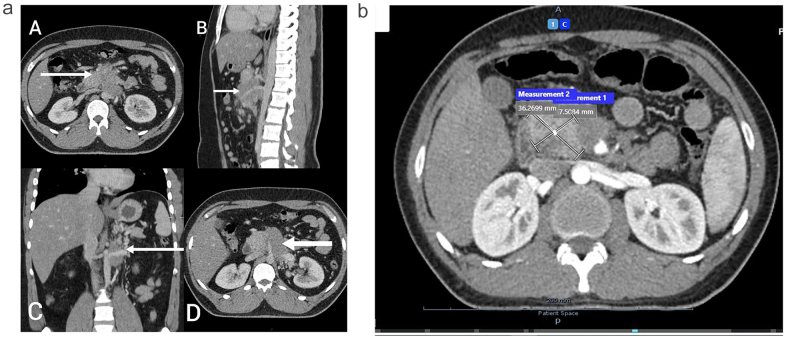
(A) Computed tomographic scan of the abdomen showing heterogeneously enhancing soft tissue density mass in the retroperitoneum. Axial views show the mass abutting the superior mesenteric vein anterolaterally (A) and inferior vena cava posteriorly (D). Lateral view showing the mass encasing the superior mesenteric artery, loss of fat planes, and inferior vena cava posteriorly (B). Frontal view showing the mass encasing the left renal vein (C). (B) Computed tomographic scan of the abdomen and pelvis showing infiltrative hypo-enhancing mass (measuring 8.1 cm in maximum oblique dimension) centered in the retroperitoneum involving the head and uncinate process of the pancreas.

The patient’s family history was unremarkable, and clinical examination showed no signs of tenderness, abdominal distension, or visceromegaly, with bowel sounds audible in all quadrants. Initial laboratory investigations, including complete blood count, renal and liver function tests, bilirubin, alkaline phosphatase, lactate dehydrogenase, and gamma-glutamyl transferase (GGT), were within normal limits, except for a mildly elevated alanine transaminase (ALT) level of 60 U/L (normal range: up to 50 U/L). A computed tomography (CT) scan of the chest, abdomen, and pelvis revealed a poorly defined, minimally enhancing hypodense mass in the retroperitoneum, closely associated with the pancreatic body and encasing major vessels, leading to their narrowing. Misty mesentery with multiple nodes/nodules suggested mesenteric panniculitis as shown in Fig. 1A.

**Figure 2:**
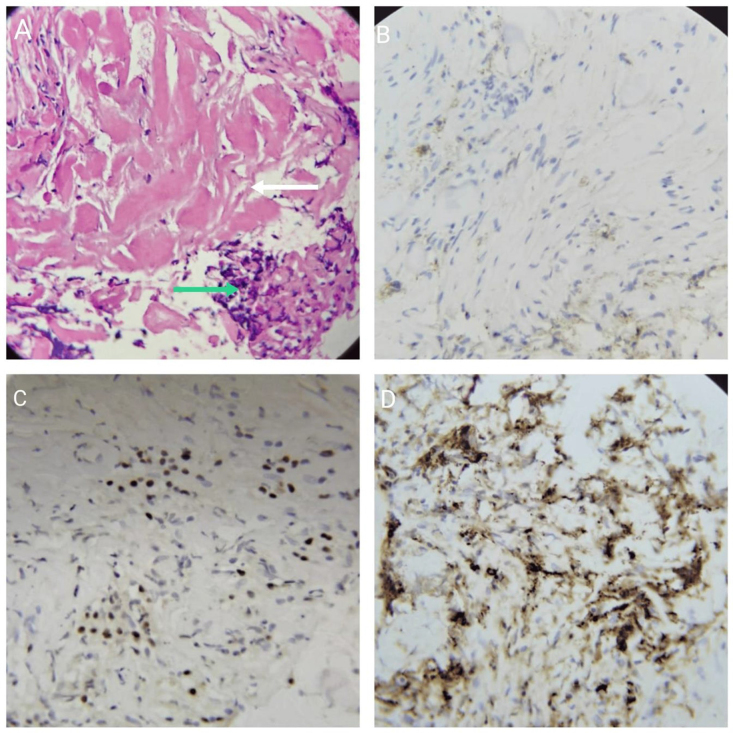
(A) EUS-guided biopsy of the pancreatic mass showing fragments of benign biliary epithelium with dense hyalinization (white arrow) and plasma cells (green arrow) (H&E, 20× obj). Immunohistochemistry CD138 (B) and immunohistochemistry MUM-1 (C) showing increased plasma cells. Immunohistochemistry highlighting IgG4-positive plasma cells (D).

Based on the radiological findings, the differential diagnosis included pancreatic cancer, tuberculosis, and lymphoma. To further investigate, an endoscopic ultrasound (EUS)-guided biopsy of the pancreatic mass was performed. The biopsy revealed fragments of benign biliary epithelium with a mild lymphoplasmacytic infiltrate, dense fibrosis, and background hemorrhage. These findings were consistent with IgG4-related retroperitoneal fibrosis. Immunohistochemistry showed plasma cells highlighted by IgG4, CD138, and MUM-1, with only a few cells staining positive for CD20 as shown in Fig. 2A.

The patient was prescribed a tablet of prednisolone 40 mg daily, a capsule of pantoprazole 40 mg daily, and oral vitamin D supplements for three months. He has adhered to the treatment regimen and has tolerated the medications well, with no reported adverse effects. The patient was instructed to follow up in one month with a CT scan of the abdomen and was advised to return to the hospital immediately if he experienced any red flag symptoms, such as fever, severe pain, or vomiting.

### Case 2 presentation

A 26-year-old male patient presented to the gastroenterology outpatient department of a tertiary care hospital with abdominal pain on and off for three years. The pain was diffuse, radiating to the back, associated with nausea, vomiting, decreased appetite, and episodic diarrhea. The pain was recently increased in intensity for one and half months and the patient gave it a score of 7 out of 10. There was no history of hematemesis, abdominal distention, weight loss, melena, or previous gastrointestinal surgeries. The patient was vitally stable with a blood pressure of 129/83 mm/Hg, pulse rate of 75 beats per minute, respiratory rate of 20 breaths per minute, and SpO2 of 99%. Abdominal examination was unremarkable for mild epigastric tenderness. Bowel sounds were audible in all four quadrants. Initial laboratory investigations were ordered and the results were shown in Table 1.

**Table 1:** Initial laboratory evaluation.

Investigation	Result	Reference range
Hemoglobin	12.8 g/dL	13-17
Total leukocyte count	14 × 10^3^ cells per microliter	4-10 × 10^3^
Platelets	196 000 cells per microliter	150-450
Serum sodium	137 mEq/L	135-145
Serum potassium	4.0 mEq/L	3.5-5.0
Serum chloride	104 mEq/L	98-108
Serum TSH	1.2 mIU/L	0.5-5
Serum calcium	8.2 mg/dl	8-10
Blood urea	36 mg/dl	8-24
Serum creatinine	1.1 mg/dl	<1.2
ALT	49 IU/L	10-50
ALP	132 IU/L	<130
Total bilirubin	1.1 mg/dl	<1.2
HBsAg	Negative	
Anti HCV (IgG)	Negative	
Stool for occult blood	Negative	
Serum amylase	48 U/L	30-110
Serum lipase	40 U/L	10-120

ALT, alanine transaminase; ALP, alkaline phosphatase; HBsAg, hepatitis surface antigen; g/dl, gram per deciliter; mEq/L, milliequivalent per liter; U/L, unit per liter; mg/dl, milligram per deciliter.

A CT scan of the abdomen and pelvis with the pancreatic protocol was done, which revealed an infiltrative hypo-enhancing mass (measuring 8.1 cm in maximum oblique dimension) centered in the retroperitoneum involving the head and uncinate process of the pancreas. Right laterally it was inseparable from the medial wall of the first part of the duodenum. Right posteriorly it was reaching up to IVC with loss of fat planes Left posterolaterally it was abuting the body of the left adrenal gland. It encased the celiac axis, common hepatic as well as a proximal splenic artery, however these vessels remain patent throughout their courses. Proximal SMV, proximal splenic vein, porto-splenic confluence as well as main portal vein were not discretely visualized, likely embedded and encased/severely attenuated by the aforementioned mass and replaced by a bunch of collaterals at porta hepatis, suggestive of cavernous transformation. Multiple adjacent and mesenteric enlarged and prominent lymph nodes were seen, and the liver was mildly enlarged and measured 17.3 cm. The spleen was mildly enlarged and measured 13.3 cm. The stomach collapsed, and atelectatic changes in the right lung middle lobe as shown in Fig. 1B. Based on the radiological findings a differential diagnosis of pancreatic cancer, retroperitoneal fibrosis, tuberculosis, and lymphoma was made. Further laboratory investigations were ordered, and the results are given in Table 2.

**Table 2:** Further laboratory evaluation.

Investigation	Result	Reference range
Serum anti transglutaminase IgA	0.57 U/ml	<2.6
Serum anti transglutaminase IgG	<0.5 U/ml	<2.6
Serum CA 19-9	6.8 U/ml	0-34
Serum CEA	0.77 ng/ml	<5
Serum IgG-4	690 mg/L	30-2010
Serum C-reactive proteins	3.02 mg/L	0-10

An EUS-guided biopsy of the pancreatic mass was done which revealed fibrin and hemorrhage. A few strips of biliary epithelium and crushed inflammatory cells were seen. Immunostains CAM5.2 is positive in epithelial strips. CD20 highlighted B lymphocytes and clusters of epithelial cells were seen. No definite evidence of malignancy was seen in the examined material. Based on the radiological and biopsy findings the patient was diagnosed as a case of IgG 4-related retroperitoneal fibrosis. The patient was started on tablet prednisolone 40 mg daily, capsule pantoprazole 40 mg daily, and oral vitamin D supplements for 3 months. The patient has been compliant with the medicine and tolerates it well, without any adverse effects. The patient was told about the advised follow-up after 1 month with a CT scan of the abdomen and was informed about red flags and to return to the hospital in case of fever, severe pain, and vomiting.

## Discussion

Retroperitoneal fibrosis (RPF) has been increasingly recognized as part of the spectrum of IgG4-related disease (IgG4-RD). RPF primarily affects men, with the average age of onset ranging between 40 and 65 years. The disease is more prevalent in males, with a male-to-female ratio of approximately 3:1. Previous research has demonstrated that IgG4-RD can involve multiple organ systems, with the retroperitoneum being a common site of manifestation.^[9]^ Other frequently affected regions include the pancreas, biliary tract, submandibular glands, major salivary glands such as the parotid, and lymph nodes.^[10,11]^ In our cases of patients diagnosed with IgG4-related RPF, predominantly involving the retroperitoneum.

RPF is classified into two subtypes: secondary and idiopathic. Idiopathic RPF accounts for approximately two-thirds of all cases, representing the majority of cases, while secondary RPF accounts for approximately one-third.^[12]^ Secondary RPF is typically associated with factors such as malignancies, inflammatory periarteritis, autoimmune diseases, retroperitoneal trauma, radiation exposure, and specific medications. The recognition of IgG4-related RPF as a clinical entity is relatively recent, and conclusive data on its morbidity and mortality are still lacking.^[11]^ A review of 13 studies of patients with IgG4-related disease found that about 10% were affected by IgG4-related RPF. Estimates suggest that retroperitoneal involvement in IgG4-RD varies between 3% and 19%. Similarly as evidenced by our cases analogs with previous literature the cause of retroperitoneal fibrosis was IgG-4 mediated.^[13]^

Diagnosing of IgG4-related retroperitoneal fibrosis (IgG4-RPF) involves clinical suspicion, radiological findings, IgG4 levels, and ultimately the gold standard tissue biopsy. Abdominal CT scans and magnetic resonance imaging (MRI) are the primary radiological tools used in this process. These imaging techniques are vital for identifying the characteristic hypertrophic, tumorous lesions typically seen in IgG4-RPF, such as those encircling arteries, the renal pelvis, ureters, or other regions of the retroperitoneum. Abdominal CT is particularly valuable in diagnosing and monitoring hydronephrosis and retroperitoneal masses.^[14]^

As both patients presented with abdominal pain, a non-specific symptom common to many gastrointestinal disorders. This symptom, combined with their histories – one patient with a B-cell lymphoproliferative disorder – raised immediate concerns for malignancy. The absence of classic symptoms such as significant weight loss, fever, or gastrointestinal bleeding added to the diagnostic ambiguity, complicating the clinical picture. A rigorous and systematic approach was taken for diagnostically, multiple potential conditions such as pancreatic cancer, tuberculosis, and lymphoma were carefully considered based on clinical presentation.

Initial CT scans revealed poorly defined, infiltrative masses in the retroperitoneum, raising differential diagnoses that included pancreatic cancer, tuberculosis, and lymphoma. The difficulty in distinguishing IgG4-RPF from these conditions arises from the similar appearance of the masses, which can involve encasement of adjacent vascular structures and surrounding tissue infiltration. In both cases, the masses exhibited characteristics suggestive of malignancy, prompting further investigation through biopsy.

To confirm the diagnosis of IgG4-RPF, histopathological evaluation was essential. EUS-guided biopsies were performed in both cases, revealing significant findings. In Case 1, the biopsy demonstrated benign biliary epithelium with lymphoplasmacytic infiltrate and dense fibrosis, alongside IgG4-positive plasma cells, which are hallmark features of IgG4-RPF. In Case 2, the biopsy indicated inflammatory changes but lacked definitive malignancy, prompting a further review of the tissue for IgG4-related features. The diagnosis was solidified by the identification of a significant number of IgG4-positive plasma cells, exceeding the threshold typically used to indicate IgG4-related disease. Ruling out malignancy was a critical aspect of the diagnostic process. Given the patients’ histories and imaging findings, the concern for pancreatic cancer and lymphoma was high. However, the presence of benign histological features, coupled with a lack of significant malignant cells in biopsy samples, allowed for the exclusion of these diagnoses.

as the definitive diagnosis often relies on the identification of characteristic histological features, such as storiform fibrosis and a marked IgG4-positive plasma cell infiltrate.

Farook *et al* reported a case that revealed a retroperitoneal tumorous mass encasing the abdominal aorta, inferior mesenteric artery, and inferior vena cava along with bilateral hydronephrosis.^[15]^ In our cases, the fibrosis mainly involved the peripancreatic area abutting the superior mesenteric artery, and inferior vena cava and renal veins. However, imaging techniques alone cannot differentiate between IgG4-related RPF and non-IgG4-RPF. The key to diagnosis lies in histopathological examination, which is crucial for ruling out benign and malignant lesions and specifically identifying inflammatory infiltrates associated with IgG4-RPF. The characteristic histopathological features of this condition include lymphocyte and plasma cell infiltrations accompanied by fibrosis, multiple lymphoid follicles, and a significant number of IgG4-positive plasma cells, typically exceeding 10 IgG4+ cells per high-power field (HPF).^[16]^ The serum IgG4 level is also a supportive diagnostic test, as more than 2.8 g/L IgG4 in serum is considered highly specific for IgG4-related diseases.^[13]^ These findings are analogous to that of other reported cases of IgG4-RPF. The serum IgG4 level can also be used as a prognostic marker, as the concentration gradually decreases with immunotherapy. However, the reference standard for diagnosing IgG4-RD involves confirmation of the histologic features such as storiform fibrosis, which is characteristic of IgG4-RPF and is more sensitive and specific than elevated serum IgG4 levels.^[17,18]^

For patients with active IgG4-related disease (IgG4-RD), glucocorticoids are generally the first-line treatment for inducing remission. This approach typically results in a reduction in blood IgG4 levels and a decrease or elimination of the mass in approximately 50% of cases.^[19]^. If the disease is uncontrolled and affects critical organs, urgent surgical intervention may be necessary to prevent irreversible damage. In terms of treatment in our cases, the use of established glucocorticoid therapy as the first-line treatment for IgG4-RD followed recognized guidelines, helping to reduce treatment bias. Potential confounders, such as other autoimmune or inflammatory disorders, were controlled through comprehensive laboratory testing, including tumor markers and autoimmune panels, to rule out alternative diagnoses. Moreover, clinical follow-up was structured to assess treatment response while monitoring for any signs that might suggest an alternative diagnosis or concurrent condition. Both patients responded well to steroids, however, their follow-up imaging is pending. For cases that are resistant or refractory, second-line treatments such as azathioprine, methotrexate, rituximab, and cyclophosphamide may be considered.^[20]^

The prognosis for idiopathic and benign forms of retroperitoneal fibrosis (RPF) is generally favorable, while malignant RPF has a poorer outlook. IgG4-RPF is a severe, aggressive form that can cause significant organ dysfunction and increased morbidity or mortality if not treated promptly. Additionally, malignancy can contribute to the development of malignant IgG4-RPF. IgG4-related disease is also linked to a heightened risk of malignancies, particularly lymphoma and pancreatic cancer. Thus, screening for cancers is crucial in the management and diagnosis of IgG4-RD.^[21]^

We acknowledge the limitations of this case series, particularly the small sample size of only two patients, which restricts the generalizability of our findings. While these cases provide important insights into the presentation, diagnosis, and management of IgG4-related retroperitoneal fibrosis in patients particularly with a history of B-cell lymphoproliferative disorder, they are insufficient for drawing broad conclusions or establishing definitive treatment protocols. We believe that further studies with larger patient cohorts are necessary to validate our observations and better understand the variability in clinical presentation and treatment responses. This case series is intended to contribute to the existing body of literature on IgG4-RD, and we encourage caution in extrapolating these results to the wider population. Explicitly discussing these limitations ensures a more transparent and cautious interpretation of our findings.

Furthermore, the follow-up period in this case series is relatively short, limited to 1 month. Unfortunately, longer-term outcomes for these patients are not yet available. However, the short follow-up was primarily due to the early stage of the treatment regimen, during which the initial response to glucocorticoid therapy was the primary focus. Given the chronic nature of IgG4-related disease and the potential for relapse, extended monitoring is essential, and we plan to continue following these patients for a more comprehensive evaluation of their long-term outcomes. In the context of this case series, the early follow-up allows us to report on the immediate therapeutic response and treatment tolerability, while highlighting the importance of ongoing observation in the management of IgG4-RD.

## Conclusion

IgG4-related disease (IgG4-RD) is a rare, chronic condition marked by inflammation and fibrosis, often presenting as retroperitoneal fibrosis (RPF). It can involve multiple organs. Diagnosis requires differentiating IgG4-related RPF from other forms and malignancies through imaging and histopathology. Glucocorticoids are the primary treatment and can induce remission and symptom relief. However, their long-term use poses risks, and alternative treatments may be necessary for cases resistant to corticosteroids. Given the potential for serious complications and an increased risk of associated malignancies, careful monitoring and follow-up are crucial. Continued research and advancements in diagnostic methods will be essential for improving the management of IgG4-RD.

## Data Availability

The datasets supporting the conclusions of this article are included within the article.

## Research registration unique identifying number (UIN)

Researchregistry (ID: 10604).
